# The support paradox: work family conflict, glass ceiling barriers, technostress, and job satisfaction among female academics in Indonesian Catholic higher education

**DOI:** 10.3389/fsoc.2026.1907341

**Published:** 2026-09-04

**Authors:** Ega Leovani, Mohamad Adam, Muhammad Ichsan Hadjri

**Affiliations:** 1 Faculty of Business and Accounting, Musi Charitas Catholic University, Palembang, Indonesia; 2 Faculty of Economics, Universitas Sriwijaya, Palembang, Indonesia

**Keywords:** female academics, glass ceiling, job satisfaction, supportive organizational culture, technostress, work–family conflict

## Abstract

**Introduction:**

Female academics face multidimensional institutional demands arising from work–family conflict, glass ceiling barriers, and technostress, which may undermine job satisfaction. Although supportive organizational culture is frequently regarded as an important organizational resource, limited evidence is available regarding its capacity to buffer the adverse associations between these demands and job satisfaction. This study examines these relationships among female academics employed in Indonesian Catholic higher education institutions.

**Methods:**

A quantitative cross-sectional survey was conducted among 406 female academics from 22 Catholic higher education institutions in Indonesia. All constructs were measured at the individual level. Partial Least Squares Structural Equation Modeling (PLS-SEM) was used to examine the associations of work–family conflict, glass ceiling barriers, and technostress with job satisfaction and to test the moderating role of perceived supportive organizational culture.

**Results:**

Work–family conflict, glass ceiling barriers, and technostress were negatively associated with job satisfaction. Perceived supportive organizational culture significantly moderated all three relationships, such that the negative associations between these institutional demands and job satisfaction became weaker at higher levels of perceived organizational support.

**Discussion:**

The findings indicate that supportive organizational culture can function as a protective resource for female academics, although such support attenuates rather than eliminates the adverse associations of role, career, and technological demands with job satisfaction. The findings extend the Job Demands–Resources framework and Conservation of Resources theory by demonstrating the buffering role of perceived organizational support among female academics in faith-based higher education.

## 1 Introduction

Higher education institutions are increasingly expected to provide supportive work environments that promote employee wellbeing, career development, and organizational sustainability. Universities frequently emphasize collegiality, inclusiveness, and employee support as fundamental institutional values designed to foster positive workplace experiences (Schein, 1992). Supportive organizational environments have generally been associated with greater employee wellbeing, organizational commitment, and job satisfaction (Rhoades and Eisenberger, 2002). Nevertheless, growing evidence indicates that female academics continue to encounter substantial occupational challenges despite working in institutions that formally promote support and inclusion (Odle-Dusseau et al., 2025; Patel et al., 2026).

Women have become an established part of the academic workforce, yet numerical participation has not produced equivalent access to senior academic and leadership positions. Indonesian higher education employed 328,241 lecturers in 2025, of whom 155,453, or 47.4%, were women. Female representation was lower at the upper ranks, accounting for approximately 42.9% of senior lecturers and 32.3% of professors. The decline across academic levels indicates that entry into the profession and advancement within it remain distinct organizational processes (Kemdiktisaintek, 2026). Similar vertical inequalities have been documented across national systems, where women become progressively less visible as authority, employment security, and institutional influence increase (Connors, 2024; Correa et al., 2025).

Among the most persistent challenges experienced by female academics are work–family conflict, glass ceiling barriers, and technostress. Academic careers require individuals to simultaneously fulfill teaching, research, community engagement, administrative, and professional development responsibilities. Such demands often overlap with family obligations, creating tensions between work and non-work domains (Greenhaus and Beutell, 1985). Female academics also continue to experience structural barriers that restrict access to leadership positions, promotion opportunities, and career advancement (Patel et al., 2026). Furthermore, the rapid digitalization of higher education has intensified technological demands, increased expectations of continuous connectivity, and contributed to growing levels of technostress among university employees (Kim and Lee, 2025; Su and Jiang, 2023).

Previous studies have consistently demonstrated that work–family conflict, glass ceiling barriers, and technostress negatively influence employee wellbeing and job satisfaction (Bai et al., 2025; Li et al., 2025; Shen et al., 2025). According to the Job Demands–Resources (JD-R) framework, excessive job demands consume employees’ physical, emotional, and psychological resources, ultimately reducing motivation and job satisfaction (Bakker and Demerouti, 2014; Demerouti, 2025). Similarly, Conservation of Resources (COR) theory suggests that individuals experience strain when valuable resources are threatened, lost, or insufficient to cope with environmental demands (Hobfoll, 1989). Consequently, multidimensional institutional demands are expected to diminish job satisfaction among female academics by generating persistent resource depletion and psychological pressure.

Although the detrimental effects of institutional demands have been widely documented, substantially less attention has been devoted to understanding whether organizational-level resources can effectively buffer these negative consequences. Existing research has predominantly focused on individual-level mechanisms such as resilience, career adaptability, self-regulation, and personal coping strategies (Bai et al., 2025; Kim and Lee, 2025). Comparatively limited evidence is available regarding the extent to which supportive organizational culture can mitigate the adverse effects of work–family conflict, glass ceiling barriers, and technostress. This omission is noteworthy because organizational culture represents a contextual resource that may shape how employees interpret, experience, and respond to workplace challenges (Schein, 1992).

The role of supportive organizational culture remains theoretically ambiguous. On the one hand, supportive cultures are expected to provide emotional support, managerial assistance, equitable treatment, and access to organizational resources that reduce workplace strain (Rhoades and Eisenberger, 2002). On the other hand, the persistence of work–family conflict, gender inequality, and technostress within institutions that publicly promote supportive values raises important questions regarding the actual effectiveness of organizational support mechanisms (Gonçalves et al., 2025; Odle-Dusseau et al., 2025). This contradiction gives rise to what may be described as the “support paradox,” whereby organizational support is highly emphasized, yet employees continue to experience significant occupational pressures and dissatisfaction.

Academic work also combines demands that arise from different parts of the employment relationship. Teaching, research, publication, student supervision, community engagement, administrative reporting, accreditation, and continuous professional development compete for time and attention. Family responsibilities may intensify this competition, particularly when work extends into evenings or weekends. Career progression can be constrained by opaque promotion processes, unequal access to sponsorship, and limited representation in decision-making. Digitalization adds a further layer through multiple platforms, rapid system changes, information overload, and expectations of immediate availability. Evidence from higher education has linked these pressures to depleted wellbeing, burnout, and weaker job attitudes (Marrinhas et al., 2023; Sakhiyya et al., 2023).

This issue is particularly relevant within faith-based higher education institutions. Catholic universities are generally associated with values of human dignity, social justice, solidarity, and employee wellbeing. Nevertheless, female academics employed in such institutions remain exposed to the same institutional pressures observed across the broader higher education sector (Patel et al., 2026). Recent studies have demonstrated that resource depletion processes continue to operate even in organizations that explicitly promote supportive and humanistic values (Demerouti, 2025; Hao et al., 2026). Consequently, it remains unclear whether supportive organizational culture genuinely functions as a protective organizational resource capable of reducing the negative effects of work–family conflict, glass ceiling barriers, and technostress on job satisfaction.

Four gaps remain. First, work–family conflict, glass ceiling barriers, and technostress have usually been examined separately, which obscures their concurrent operation across role, career, and digital domains. Second, organizational support has often been treated as a direct predictor, while its capacity to alter demand–satisfaction relationships has received less integrated testing. Third, evidence on female academics in Indonesian Catholic institutions remains limited despite the tension between humanistic institutional values and gendered academic work. Fourth, studies with strong in-sample measurement results rarely examine whether the estimated model also performs well outside the estimation sample. The present analysis addresses these gaps by integrating the three demands, specifying perceived supportive organizational culture as a moderator, and evaluating explanatory as well as predictive performance.

Two research questions guide the analysis. RQ1 asks how work–family conflict, glass ceiling barriers, and technostress are associated with job satisfaction among female academics in Indonesian Catholic higher education. RQ2 asks to what extent perceived supportive organizational culture weakens the negative associations between these demands and job satisfaction. The proposed contribution is therefore conceptual, contextual, and methodological: a bounded interpretation of the support paradox is developed, the model is tested across 22 Catholic institutions, and the strength of the moderation effects is assessed alongside out of-sample prediction.

### 1.1 The support paradox in higher education

Catholic higher education provides a theoretically demanding setting for examining these conditions. Catholic universities are normatively oriented toward human dignity, solidarity, justice, and the integral development of employees and students (Pope John Paul II, 1990). Support for employee wellbeing should therefore be embedded not only in interpersonal relationships but also in institutional arrangements. A contradiction arises when supportive values and positive perceptions of organizational culture coexist with persistent role conflict, career barriers, and digital strain. The term support paradox is used here as an analytical label for that coexistence. It does not constitute a separate theory and does not imply that organizational support is ineffective. Rather, it focuses attention on the boundary between support that moderates adverse experiences and organizational reform that removes their sources.

The Job Demands Resources (JD R) framework and Conservation of Resources (COR) theory provide complementary explanations for this boundary. JD–R theory classifies sustained role, career, and technological pressures as demands because they require continued cognitive, emotional, or temporal effort. Supportive policies, fair treatment, useful feedback, development opportunities, and flexible work arrangements function as resources when they facilitate task completion or reduce the costs of demanding work (Bakker and Demerouti, 2007, 2014; Demerouti, 2025). COR theory explains why repeated demands become consequential: strain is produced when valued resources are threatened, depleted, or insufficiently replenished (Hobfoll, 1989). Organizational support may slow resource loss, but a resource does not automatically remove the structural or technological condition that initiated the loss.

Supportive organizational culture refers to shared and enacted organizational arrangements through which employee contributions are recognized, relevant information is communicated, work and non-work responsibilities are accommodated, professional development is supported, and employee wellbeing is treated as a legitimate organizational concern. The construct is related to perceived organizational support, but it is broader than a general belief that the institution values an employee. It concerns the perceived cultural consistency of communication, recognition, flexibility, career development, and commitment to employees (Eisenberger et al., 1986; Rhoades and Eisenberger, 2002; Schein, 1992). Because the present data were collected from individuals, the construct represents perceived culture rather than an independently measured organizational property.

JD R theory predicts that job resources can reduce the health-impairment consequences of high demands. COR theory specifies the mechanism: supportive practices protect time, energy, status, information, and development opportunities that would otherwise be lost. Neither theory requires a resource to neutralize a demand. Buffering can therefore be statistically significant while remaining substantively incomplete. This distinction is central to the support paradox. A positive interaction coefficient may indicate that supportive culture reduces the steepness of a negative demand–satisfaction association, whereas a remaining negative conditional slope indicates that the demand persists. The empirical question is not whether support is simply present, but how far its protective effect extends.

### 1.2 Work–family conflict and job satisfaction

Work–family conflict refers to a form of inter-role conflict in which pressures associated with work and family domains become mutually incompatible (Greenhaus and Beutell, 1985). Academic careers require substantial commitments to teaching, research, publication, supervision, and administrative responsibilities. Simultaneously, female academics frequently assume significant caregiving and family responsibilities. The coexistence of these competing demands may create role overload, emotional exhaustion, and psychological strain.

Meta-analytic evidence has consistently linked work family conflict to lower job and life satisfaction, poorer psychological health, and stronger withdrawal intentions (Amstad et al., 2011). Among female university teachers, institutional expectations of uninterrupted professional commitment may be combined with culturally gendered expectations surrounding caregiving and domestic responsibility, increasing competition for time and recovery (Sakhiyya et al., 2023). Under COR theory, repeated transfers of time and emotional energy from family to work reduce the resources available for both domains. Satisfaction with academic work is consequently expected to decline when work is experienced as a continuing source of family disruption.

*H1*: Work–family conflict negatively affects job satisfaction.

### 1.3 Glass ceiling and job satisfaction

The glass ceiling denotes a pattern of inequality that becomes more pronounced at higher organizational levels and cannot be fully explained by job-relevant characteristics (Cotter et al., 2001). In academic organizations, the barrier may be reproduced through restricted access to influential networks, unequal allocation of strategic assignments, slower promotion, underrepresentation in leadership, and decision criteria that appear formally neutral but reward historically masculine career patterns. Gendered-organization theory further explains that jobs, authority structures, and standards of the ideal worker are not institutionally neutral; organizational routines can embed assumptions about availability, mobility, and care responsibilities (Acker and Wagner, 2019).

Glass ceiling perceptions affect satisfaction because promotion and leadership access signal whether present contributions are likely to produce future recognition. Women who observe unequal advancement despite comparable qualifications may interpret the employment relationship as procedurally unfair and may discount the value of continued investment. Studies of higher education have documented persistent barriers in recruitment, promotion, leadership, and secure academic employment, including in systems where equality is formally endorsed (De Angelis et al., 2021). From a COR perspective, the barrier threatens future resources such as status, income, autonomy, and influence. Job satisfaction should therefore be lower when career mobility is perceived as structurally constrained.

*H2*: Glass ceiling negatively affects job satisfaction.

### 1.4 Technostress and job satisfaction

Technostress is strain generated when technological characteristics and digital work requirements exceed the resources available for adaptation. Techno-overload increases the volume or speed of work; techno-invasion extends work into private time; techno-complexity creates skill and learning demands; techno-insecurity produces concern about technological displacement or comparative competence; and techno-uncertainty requires repeated adjustment to changing systems (Ragu-Nathan et al., 2008; Tarafdar et al., 2007). These dimensions are increasingly relevant to academic work because teaching platforms, research systems, performance dashboards, administrative portals, and messaging applications are used simultaneously.

The problem is not reducible to low digital competence. Technostress can also arise when platforms duplicate tasks, procedures are unclear, systems change without adequate consultation, and communication norms require near-continuous responsiveness. Studies of university employees have associated these conditions with burnout, impaired wellbeing, and reduced satisfaction (Marrinhas et al., 2023; Wang and Li, 2019). JD R theory classifies such demands as resource-consuming because attention is redirected from substantive academic work toward system navigation, troubleshooting, and monitoring. Satisfaction is expected to decline when technology increases rather than reduces the effort required to perform academic responsibilities.

*H3*: Technostress negatively affects job satisfaction.

### 1.5 Supportive organizational culture as a buffering resource

Supportive culture may weaken work family conflict by legitimizing flexibility, limiting after-hours demands, and allowing workload adjustments when family responsibilities intensify. These practices protect recovery time and reduce the penalty associated with temporary constraints on availability. The resource does not remove all competing roles, but it can prevent institutional procedures from amplifying the conflict. A weaker negative association is therefore expected where work life flexibility and managerial responsiveness are perceived as culturally consistent.

*H4*: Perceived supportive organizational culture moderates the relationship between work–family conflict and job satisfaction, such that the negative association is weaker at higher levels of perceived support.

Glass ceiling barriers may also become less damaging when promotion criteria are communicated clearly, contributions are recognized, mentoring is available, and career-development support can be accessed without dependence on informal sponsorship. These resources preserve procedural trust and professional agency even when structural inequalities have not been fully removed. Yet support cannot be equated with equal outcomes; a moderation effect would indicate attenuation of the adverse association rather than proof that the barrier no longer exists.

*H5*: Perceived supportive organizational culture moderates the relationship between glass ceiling barriers and job satisfaction, such that the negative association is weaker at higher levels of perceived support.

**Figure 1:**
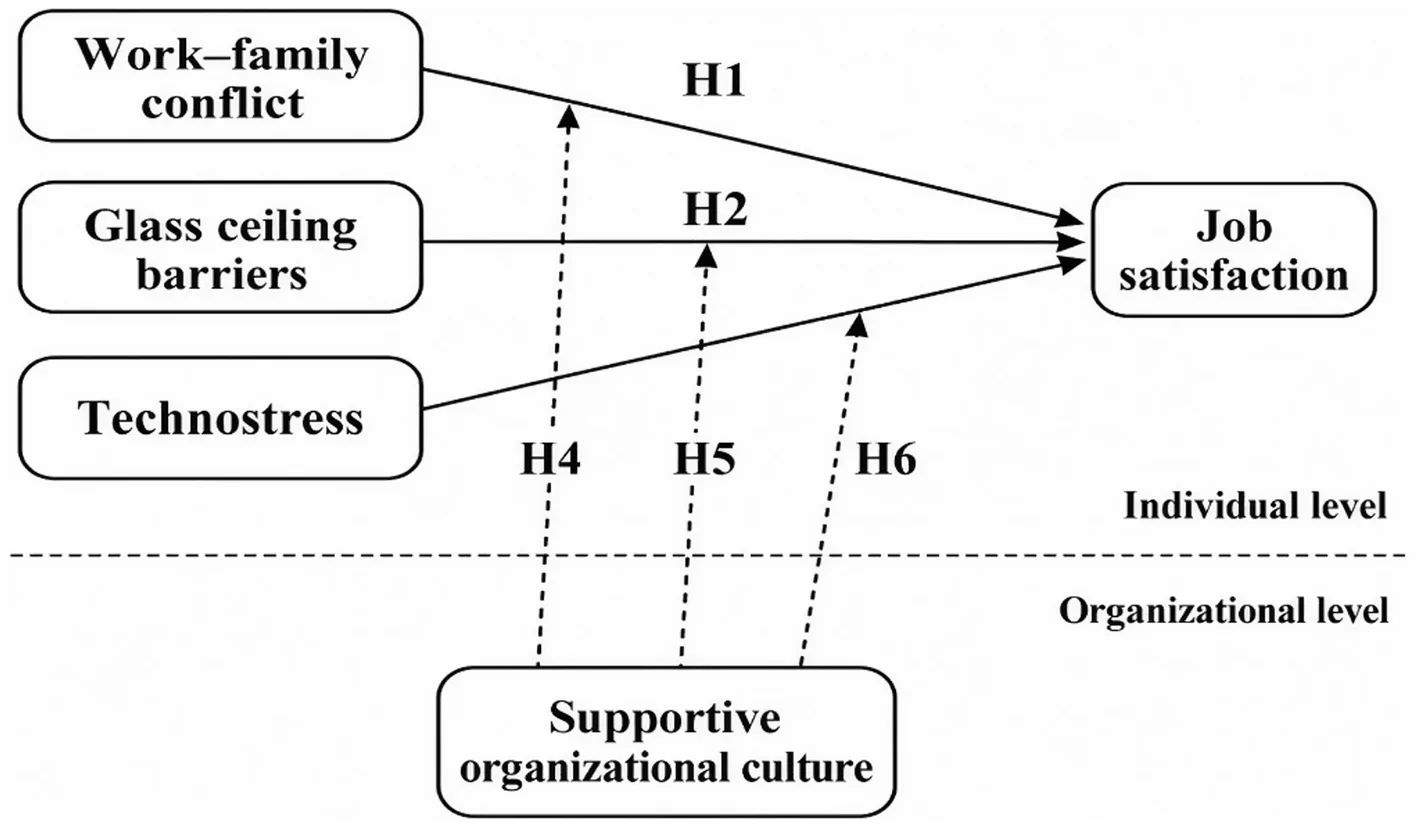
Individual-level conceptual model. Solid arrows represent direct associations; dashed boxes and arrows represent the interaction terms used to test moderation. Source: Developed by the authors based on the Job Demands–Resources (JD-R) framework (Bakker and Demerouti, 2007) and Conservation of Resources (COR) theory (Hobfoll, 1989).

Technostress can be buffered when digital changes are communicated in advance, technical assistance is accessible, learning time is protected, and norms constrain communication outside working hours. Such resources reduce uncertainty and prevent digital demands from consuming unrestricted amounts of attention and recovery time. The protective effect should be strongest when support is aligned with the source of technological strain rather than expressed only as general concern for employee welfare (see Figure 1).

*H6*: Perceived supportive organizational culture moderates the relationship between technostress and job satisfaction, such that the negative association is weaker at higher levels of perceived support.

## 2 Materials and methods

A quantitative cross-sectional survey was used to estimate associations among perceived working conditions. The design does not establish temporal order or causal effects; causal terminology is therefore avoided in the interpretation of the results. The unit of analysis was the individual female academic. All constructs, including supportive organizational culture, were measured through individual responses. No institutional mean was calculated, and the culture measure was not specified as an organization-level variable. Data were collected online between January and March 2025. Participation was voluntary, and the questionnaire could be accessed only after electronic informed consent was provided. A seven-point response scale was used, ranging from 1 (strongly disagree) to 7 (strongly agree). Anonymous participation and the removal of identifying information were used to reduce evaluation apprehension and protect confidentiality.

The target population comprised female academics employed by Indonesian higher education institutions affiliated with the Association of Catholic Higher Education Institutions (APTIK). A nonprobability criterion-based purposive strategy was combined with proportional recruitment targets across participating institutions and academic ranks. The criterion component ensured that respondents held an active academic appointment, participated in academic work, and routinely used institutional digital systems. Recruitment within the defined categories was not random; the procedure should therefore not be described as probability stratified sampling. This clarification distinguishes eligibility screening and recruitment targets from random selection (Campbell et al., 2020). Valid responses were obtained from 406 academics across 22 institutions. Institutional contributions ranged from one to 50 respondents, and the five largest contributing institutions accounted for 51.7% of the sample. The observations were therefore nested within institutions, but the structural model was estimated at the individual level without cluster-robust standard errors or a multilevel specification. This limitation is considered when the precision and generalizability of the estimates are interpreted (see Tables 1, 2).

**Table 1:** Distribution of respondents across participating Catholic higher education institutions (*N* = 406).

Institution	*n*	%
Universitas Atma Jaya Jakarta	44	10.8
Universitas Katolik Parahyangan	47	11.6
Universitas Sanata Dharma	18	4.4
Universitas Atma Jaya Yogyakarta	50	12.3
Universitas Katolik Soegijapranata	37	9.1
Universitas Widya Mandala	29	7.1
Universitas Katolik Widya Karya	32	7.9
Universitas Atma Jaya Makassar	18	4.4
Universitas Katolik Widya Mandira	7	1.7
Universitas Katolik St. Thomas Sumatera Utara	7	1.7
Universitas Katolik Musi Charitas	28	6.9
STIKes Sint Carolus	20	4.9
Universitas De La Salle Manado	4	1.0
Universitas Widya Dharma Pontianak	6	1.5
Universitas Katolik Darma Cendika	6	1.5
STIKes Katolik St. Vincentius A Paulo	16	3.9
STIKes Stella Maris Makassar	11	2.7
STIKes Gunung Maria Tomohon	7	1.7
Universitas Katolik Weetibula	4	1.0
STIKes Santa Elisabeth Medan	8	2.0
Universitas Santo Borromomeus	1	0.2
STIKes Panti Rapih	6	1.5
Total	406	100.0

The five largest contributing institutions accounted for 51.7% of the sample. The structural model remained an individual-level model and did not use cluster-robust or multilevel standard errors.

**Table 2:** Participant characteristics (*N* = 406).

Characteristic	Category	n	%
Age	≤30 years	110	27.1
	31–45 years	141	34.7
	46–60 years	89	21.9
	>60 years	66	16.3
Employment status	Permanent	310	76.4
	Contract	96	23.6
Institutional tenure	0–5 years	188	46.3
	6–10 years	181	44.6
	11–20 years	37	9.1
Highest qualification	Bachelor	7	1.7
	Master	357	87.9
	Doctoral/specialist	42	10.3
Academic rank	Assistant professor (Asisten Ahli)	192	47.3
	Associate professor (Lektor)	165	40.6
	Senior associate professor (Lektor Kepala)	36	8.9
	Professor	13	3.2
Time in current rank	<1 year	8	2.0
	1–3 years	275	67.7
	4–6 years	73	18.0
	7–10 years	47	11.6
	>10 years	3	0.7
Marital status	Single	39	9.6
	Married	360	88.7
	Formerly married	7	1.7
Dependents	None	43	10.6
	1–2	307	75.6
	3–4	52	12.8
	>4	4	1.0

Report participant characteristics before the measurement subsection so that the sample composition and limits of generalizability are transparent.

### 2.1 Measurement and operationalization of variables

The questionnaire was prepared in Indonesian. Items were adapted from established conceptual and measurement sources, reviewed by subject-matter experts for language and contextual fit, and pilot-tested before the main survey. Adaptation was required because several original measures were developed for general employment settings and needed wording that reflected academic promotion, teaching and research responsibilities, institutional digital systems, and Catholic higher education. The full item wording is retained in the study instrument; Table 3 reports the construct definitions, dimensions, number of items, and primary sources needed to evaluate content coverage.

**Table 3:** Measurement specification.

Construct	Definition	Dimensions	Items	Primary source(s)
Work–family conflict	Inter-role incompatibility in which work impedes family participation.	Time-based conflict; strain-based conflict; behavior-based conflict	26	Greenhaus and Beutell (1985)
Glass ceiling barriers	Perceived structural inequality that restricts advancement and becomes more pronounced at higher organizational levels.	Inequality at higher levels; increasing gender gap over time; control for job-relevant characteristics; inequality in promotion and compensation	25	Cotter et al. (2001)
Technostress	Strain associated with the demands and consequences of digital technology use at work.	Techno-overload; techno-complexity; techno-insecurity; techno-invasion; techno-uncertainty	24	Tarafdar et al. (2007) and Ragu-Nathan et al. (2008)
Perceived supportive organizational culture	Perceived consistency of organizational values and practices that protect employee development and wellbeing.	Organizational communication; employee recognition; work–life flexibility; career development support; commitment to employees	48	Eisenberger et al. (1986), Rhoades and Eisenberger (2002), and Schein and Schein (2017)
Job satisfaction	Evaluative judgment of major extrinsic and intrinsic aspects of academic employment.	Salary; promotion; supervision; benefits; contingent rewards; operating procedures; coworkers; nature of work; communication	27	Spector (1985)

All constructs were estimated as reflective higher-order components using a two-stage approach. First-order dimensions were estimated from their items, after which dimension scores were used as indicators of the corresponding higher-order construct. Consequently, the reported higher-order loadings and AVE values describe the relationship between each broad construct and its dimensions; they should not be interpreted as item-level correlations. Perceived supportive organizational culture was measured at the individual level and was not aggregated across respondents within an institution.

### 2.2 Statistical analysis framework

Data analysis was conducted using Partial Least Squares Structural Equation Modeling (PLS-SEM) with SmartPLS 4 software. PLS-SEM was selected because of its suitability for examining complex relationships involving latent constructs and moderating effects (Hair et al., 2010; Sarstedt et al., 2021). Partial least squares structural equation modeling was performed in SmartPLS 4. PLS–SEM was selected because the model combines reflective higher-order constructs and interaction terms and because predictive performance was examined alongside explanatory relationships (Hair and Alamer, 2022; Hair and Sarstedt, 2021). The higher-order components were estimated with a two-stage procedure. Indicator and dimension reliability, internal consistency, convergent validity, discriminant validity, and collinearity were examined before the structural paths were interpreted.

Path significance was evaluated with 5,000 bootstrap samples. Standardized coefficients, t values, *p* values, and bias-corrected 95% confidence intervals are reported. The moderation terms were created between mean-centered construct scores. Conditional effects were calculated at one standard deviation below the mean, at the mean, and one standard deviation above the mean of perceived supportive organizational culture. Explanatory power was assessed through *R*^2^, whereas out-of-sample prediction was evaluated with PLSpredict by comparing PLS–SEM prediction errors with a linear-model benchmark (Shmueli et al., 2019). Several procedural and statistical checks were used to address common method concerns. Participation was anonymous, items were framed as perceptions rather than evaluations of identifiable supervisors, and respondents were informed that no institution-level result would identify an individual. Full-collinearity VIF values below 3.3 were treated as a diagnostic against severe common method inflation (Kock, 2015). This diagnostic cannot exclude common method bias, particularly because all variables were self-reported in a single survey.

### 2.3 Ethics policies in human research

Ethical approval for this study was obtained from the Health Research Ethics Committee of Universitas Prima Indonesia under reference number 033/KEPK/UNPRI/XI/2024. Before data collection. The committee is institutionally separate from the authors’ home universities and served as an external reviewer of the multisite survey protocol. Electronic informed consent was obtained from all participants. No personal identifiers were retained, and withdrawal was permitted at any point before questionnaire submission. Participation was voluntary, and informed consent was obtained electronically before respondents accessed the questionnaire. Confidentiality and anonymity were maintained throughout the study by removing personal identifiers from the dataset. Respondents were informed of their right to withdraw from participation at any stage without penalty.

## 3 Results

**Figure 2:**
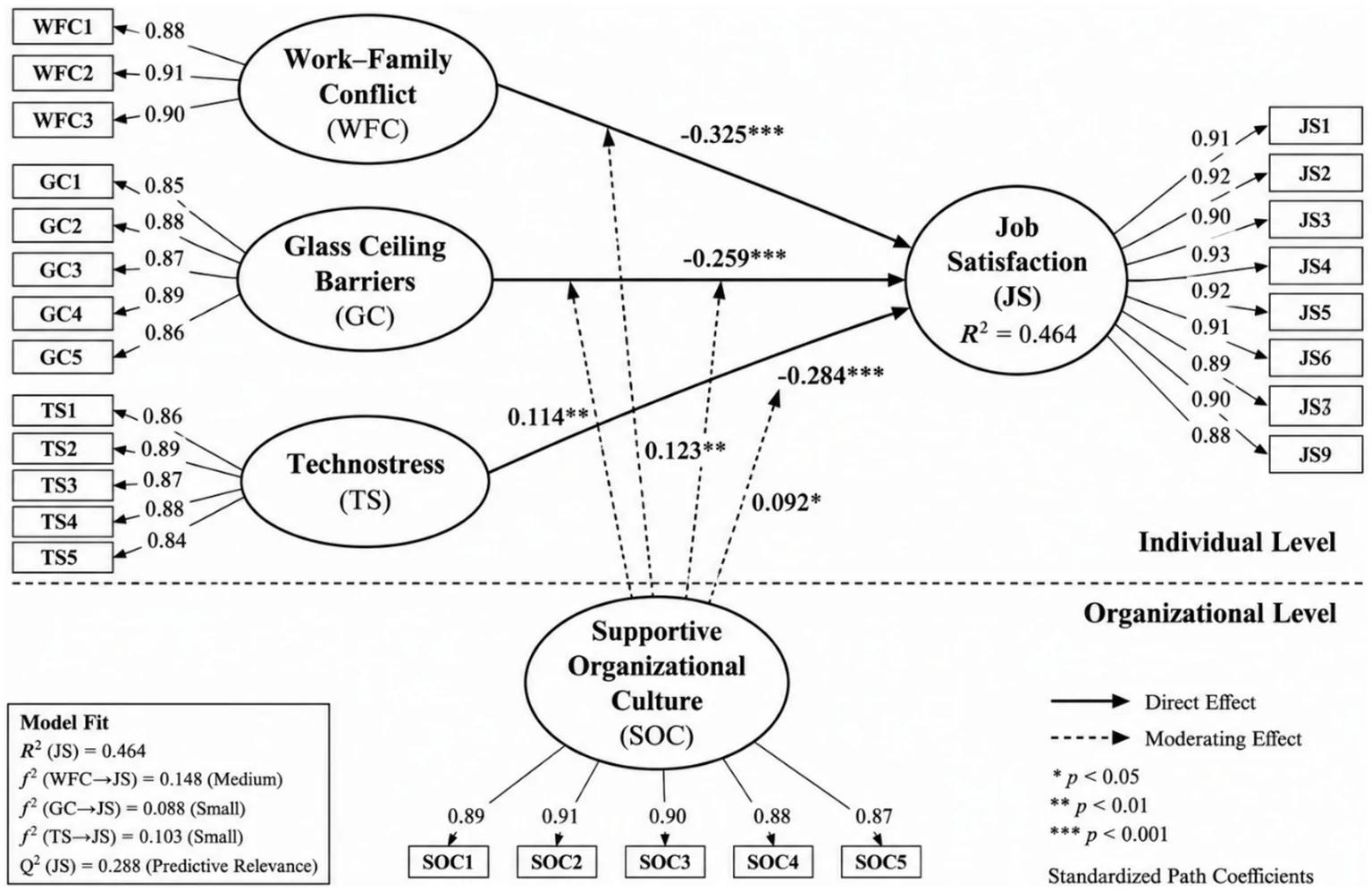
Measurement and structural model assessment showing the relationships among work–family conflict, glass ceiling, technostress, supportive organizational culture, and job satisfaction. Source: Primary Data Processing (SmartPLS 4, 2025).

The proposed research model was analyzed using Partial Least Squares Structural Equation Modeling (PLS-SEM) using SmartPLS 4. The analysis was conducted in two stages. The first stage involved the evaluation of the measurement model to assess indicator reliability, internal consistency reliability, convergent validity, and discriminant validity. The second stage focused on the structural model assessment to examine the proposed relationships and the moderating role of supportive organizational culture. The estimated research model is illustrated in Figure 2.

All higher-order dimension loadings exceeded 0.80. Cronbach’s alpha ranged from 0.959 to 0.993, composite reliability from 0.971 to 0.994, and AVE from 0.832 to 0.977. The HTMT ratios ranged from 0.040 to 0.405, and all predictor VIF values were below 3.3. These results support internal consistency, convergent validity, discriminant validity, and the absence of severe collinearity. Table 4 reports values for each construct rather than pooled ranges. The reliability estimates require qualified interpretation. Alpha and composite reliability values above 0.95, together with AVE values close to one, indicate highly homogeneous dimensions and may also signal conceptual overlap or redundant content. The two-stage higher-order specification partly explains the ceiling values because broad constructs were represented by strongly correlated dimension scores rather than by raw items. Nevertheless, the results do not justify treating high reliability as unconditionally superior. Redundancy remains plausible, especially for supportive organizational culture and work–family conflict, and future scale refinement should test whether shorter measures preserve content coverage while improving discrimination among dimensions. Low HTMT ratios and acceptable VIF values reduce concerns about overlap between different constructs but do not eliminate redundancy within a construct or common method bias.

**Table 4:** Higher-order measurement model results.

Construct	Dimension loadings	*α*	CR	AVE
Work–family conflict	0.986–0.991	0.988	0.992	0.977
Glass ceiling barriers	0.853–0.975	0.959	0.971	0.893
Technostress	0.970–0.984	0.989	0.991	0.958
Perceived supportive organizational culture	0.983–0.989	0.993	0.994	0.972
Job satisfaction	0.801–0.948	0.974	0.978	0.832

α, Cronbach’s alpha; CR, composite reliability; AVE, average variance extracted. HTMT = 0.040–0.405; all structural predictor VIF values < 3.3. Extremely high reliability should be interpreted cautiously because it may indicate homogeneous or redundant dimension content.

Discriminant validity was verified using both the Fornell-Larcker criterion and the Heterotrait-Monotrait ratio (HTMT). The Fornell-Larcker criterion was satisfied for all constructs, while HTMT values ranged from 0.040 to 0.405, remaining well below the conservative threshold of 0.85. These results confirm that the constructs are empirically distinct and capture different theoretical concepts. Furthermore, all variance inflation factor (VIF) values remained below the recommended threshold of 3.3, indicating the absence of multicollinearity issues. The coefficient of determination (R^2^) for job satisfaction was 0.464, suggesting that work–family conflict, glass ceiling, technostress, supportive organizational culture, and the interaction effects collectively explained 46.4% of the variance in job satisfaction. Overall, the results support the adequacy of the measurement model and justify proceeding to the structural model assessment and hypothesis testing.

**Figure 3:**
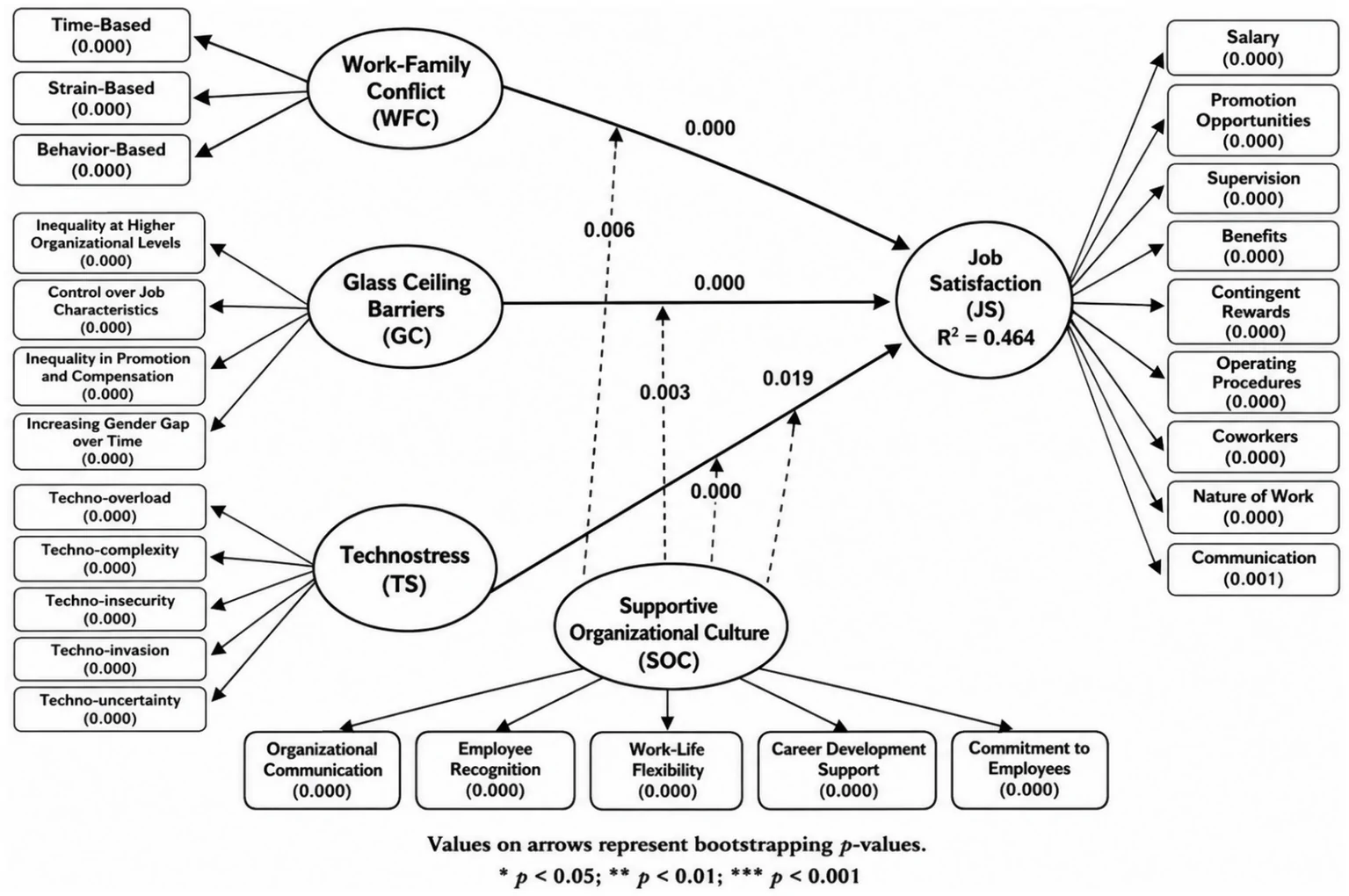
Structural model assessment showing the effects of work–family conflict, glass ceiling, and technostress on job satisfaction, as well as the moderating role of supportive organizational culture. Source: Primary Data Processing (SmartPLS 4, 2026).

The estimated model is presented in Figure 2, and the bootstrap results are reported in Table 5. Work–family conflict showed the largest negative association with job satisfaction [*β* = −0.325, 95% CI (−0.392, −0.249)], followed by technostress [*β* = −0.284, 95% CI (−0.354, −0.213)] and glass ceiling barriers [*β* = −0.259, 95% CI (−0.330, −0.183)]. The confidence intervals excluded zero, supporting H1–H3. Perceived supportive organizational culture was positively associated with job satisfaction [*β* = 0.216, 95% CI (0.138, 0.294)]. All three interaction terms were positive and significant: culture × work–family conflict [*β* = 0.114, 95% CI (0.028, 0.192)], culture × glass ceiling [*β* = 0.123, 95% CI (0.040, 0.202)], and culture × technostress [*β* = 0.092, 95% CI (0.016, 0.172)]. H4–H6 were therefore supported. The positive signs indicate attenuation because the corresponding direct associations were negative (see Figure 3).

**Table 5:** Structural path estimates and hypothesis tests.

Hyp.	Path	*β*	*t*	*p*	BC 95% CI	Decision
H1	Work–family conflict - job satisfaction	−0.325	8.864	<0.001	[−0.392, −0.249]	Supported
H2	Glass ceiling barriers - job satisfaction	−0.259	6.899	<0.001	[−0.330, −0.183]	Supported
H3	Technostress - job satisfaction	−0.284	7.902	<0.001	[−0.354, −0.213]	Supported
H4	Culture × work–family conflict - job satisfaction	0.114	2.747	0.006	[0.028, 0.192]	Supported
H5	Culture × glass ceiling - job satisfaction	0.123	2.956	0.003	[0.040, 0.202]	Supported
H6	Culture × technostress - job satisfaction	0.092	2.339	0.019	[0.016, 0.172]	Supported
—	Perceived supportive organizational culture - job satisfaction	0.216	5.463	<0.001	[0.138, 0.294]	Ancillary

BC 95% CI = bias-corrected 95% bootstrap confidence interval based on 5,000 resamples. The main effect of perceived supportive organizational culture is reported as an ancillary path.

The structural model assessment was conducted to examine whether multidimensional institutional demands significantly influence the job satisfaction of female academics and whether supportive organizational culture functions as an effective organizational resource capable of mitigating these adverse effects. Following the Job Demands–Resources (JD-R) framework and Conservation of Resources (COR) theory, the analysis evaluated both the direct effects of work–family conflict, glass ceiling, and technostress on job satisfaction and the interaction effects involving supportive organizational culture.

A bootstrapping procedure with 5,000 resamples was employed to assess the statistical significance of the proposed relationships. The evaluation was based on standardized path coefficients (β), t-statistics, and *p*-values. Significant interaction terms indicate that supportive organizational culture changes the strength of the relationships between institutional demands and job satisfaction, thereby providing empirical evidence regarding the existence of the support paradox. The results of the structural model assessment and hypothesis testing are presented in Table 6.

**Table 6:** Conditional associations between demands and job satisfaction at different levels of perceived supportive organizational culture.

Demand	Level of perceived support	Conditional *β*
Work–family conflict	Low (−1 SD)	−0.439
	Mean	−0.325
	High (+1 SD)	−0.211
Glass ceiling barriers	Low (−1 SD)	−0.382
	Mean	−0.259
	High (+1 SD)	−0.137
Technostress	Low (−1 SD)	−0.376
	Mean	−0.284
	High (+1 SD)	−0.192

All conditional coefficients remained negative at high support. This is the central evidence that support attenuated but did not eliminate the adverse associations.

The results presented in Table 5 indicate that all proposed hypotheses were supported. Work–family conflict exerted a significant negative effect on job satisfaction (*β* = −0.325, *t* = 8.864, *p* < 0.001), supporting H1. This finding suggests that increasing incompatibility between work and family responsibilities is associated with lower levels of job satisfaction among female academics. Among the direct effects examined, work–family conflict demonstrated the strongest negative influence on job satisfaction, highlighting the importance of balancing professional and family roles within academic careers.

Glass ceiling barriers were also found to negatively affect job satisfaction (*β* = −0.259, *t* = 6.899, *p* < 0.001), supporting H2. The result indicates that perceptions of limited career advancement opportunities, gender-based barriers, and organizational inequality reduce positive evaluations of the work environment. Female academics who perceive stronger glass ceiling barriers are more likely to experience lower levels of job satisfaction. Similarly, technostress exerted a significant negative effect on job satisfaction (*β* = −0.284, *t* = 7.902, *p* < 0.001), supporting H3. This finding suggests that excessive technological demands, continuous digital connectivity, and increasing technology-related complexity contribute to reduced satisfaction with academic work. The result further indicates that digital transformation may create unintended psychological burdens for female academics.

The moderating effects of supportive organizational culture were found to be significant across all proposed relationships. Supportive organizational culture significantly moderated the relationship between work–family conflict and job satisfaction (*β* = 0.114, *t* = 2.747, *p* = 0.006), supporting H4. The positive interaction coefficient indicates that supportive organizational culture weakens the negative effect of work–family conflict on job satisfaction. In supportive institutional environments, the adverse consequences of competing work and family demands become less pronounced. Supportive organizational culture also moderated the relationship between glass ceiling and job satisfaction (*β* = 0.123, *t* = 2.956, *p* = 0.003), supporting H5. The positive interaction effect suggests that supportive organizational practices, inclusive values, and equitable treatment help reduce the detrimental impact of perceived career barriers on job satisfaction. Female academics experiencing supportive organizational environments are therefore less negatively affected by glass ceiling perceptions.

Supportive organizational culture significantly moderated the relationship between technostress and job satisfaction (*β* = 0.092, *t* = 2.339, *p* = 0.019), supporting H6. The result indicates that organizational support, leadership flexibility, and collaborative workplace relationships mitigate the adverse effects of technological pressures on employee satisfaction. Consequently, supportive organizational culture functions as an important organizational resource that buffers the negative influence of multidimensional institutional demands on the job satisfaction of female academics.

Conditional effects clarify the magnitude of the moderation. At one standard deviation above the mean of perceived supportive organizational culture, all three demand–satisfaction slopes remained negative: −0.211 for work–family conflict, −0.137 for glass ceiling barriers, and −0.192 for technostress. Support therefore reduced the estimated adverse association but did not neutralize it. The interaction coefficients were also smaller than the corresponding direct coefficients. Their absolute magnitudes were approximately 35% of the work–family coefficient, 48% of the glass ceiling coefficient, and 32% of the technostress coefficient. These ratios are descriptive comparisons rather than formal effect-size tests, but they demonstrate why statistical confirmation should not be interpreted as complete protection.

Following the assessment of the structural relationships, the model’s explanatory power was evaluated using the coefficient of determination (*R*^2^) and adjusted *R*^2^. These values indicate the proportion of variance in job satisfaction explained by work–family conflict, glass ceiling barriers, technostress, perceived supportive organizational culture, and the interaction terms (see Tables 7, 8).

**Table 7:** Coefficient of determination.

Endogenous construct	*R* ^2^	Adjusted *R*^2^	Interpretation
Job satisfaction	0.464	0.454	Moderate explanatory power

**Table 8:** PLSpredict assessment results.

Criterion	Result	Interpretation
*Q*^2^predict	All indicators > 0 (0.273–0.397)	Predictive relevance established
PLS-SEM vs. LM (RMSE)	Majority of PLS-SEM RMSE values higher than LM RMSE	Limited predictive power
PLS-SEM vs. LM (MAE)	Majority of PLS-SEM MAE values higher than LM MAE	Limited predictive power
Overall Assessment	Predictive relevance established	Low predictive power

Source: Primary Data Processing (SmartPLS 4, 2026).

The coefficient of determination indicates that work–family conflict, glass ceiling barriers, technostress, perceived supportive organizational culture, and the interaction terms collectively explain 46.4% of the variance in job satisfaction. The adjusted *R*^2^ value of 0.454 indicates that the model retains moderate explanatory power after accounting for the number of predictors. The remaining 53.6% of the variance is explained by factors not included in the present model.

The model explained 46.4% of the variance in job satisfaction (*R*^2^ = 0.464). This value represents moderate explanatory power, but it also means that 53.6% of the variance remained unexplained. Compensation arrangements, leadership quality, psychological safety, employment security, disciplinary norms, institutional resources, personal values, and non-work support may account for part of the remaining variance. All *Q*^2^predict values were positive and ranged from 0.273 to 0.397, indicating that predictions from the PLS–SEM model improved on a naive mean-based benchmark. Most PLS–SEM RMSE and MAE values, however, were higher than those from the linear model. The model therefore showed predictive relevance but limited comparative predictive power. Strong measurement properties and statistically significant paths do not guarantee superior out-of-sample prediction. The present model is more defensible as an explanatory account of conditional associations than as a stand-alone forecasting tool.

## 4 Discussion

The findings demonstrated that work–family conflict, glass ceiling barriers, and technostress significantly reduced the job satisfaction of female academics. Furthermore, supportive organizational culture significantly moderated all three relationships, indicating that organizational support weakened the negative effects of multidimensional institutional demands on job satisfaction. These findings provide empirical support for the Job Demands–Resources (JD-R) framework and Conservation of Resources (COR) theory, suggesting that excessive job demands deplete valuable psychological resources, whereas organizational resources help employees cope with workplace pressures and preserve wellbeing (Bakker and Demerouti, 2007; Hobfoll, 1989). The results are consistent with recent studies reporting that increasing workplace pressures continue to diminish employee wellbeing, job satisfaction, and psychological functioning across higher education institutions and knowledge-intensive professions (Bai et al., 2025; Hao et al., 2026; Shen, 2026).

The three negative coefficients show that dissatisfaction was associated with pressures located in different parts of academic employment. Work–family conflict had the largest direct coefficient. This result is consistent with an occupation in which time boundaries are porous and professional performance can be maintained by transferring work into periods otherwise available for care, rest, or family interaction. The cost is not limited to fewer hours at home. Strain and work-oriented behavior can remain present after a task has ended, which helps explain why role conflict is strongly related to the evaluation of the job itself. COR theory is supported because the association is compatible with continuing losses of time, emotional availability, and recovery capacity (Amstad et al., 2011; Regina and Allen, 2023). The findings are also aligned with recent studies demonstrating that female academics remain disproportionately affected by role overload, emotional exhaustion, and competing professional and domestic responsibilities (Odle-Dusseau et al., 2025; Patel et al., 2026; Hao et al., 2026). Consistent with COR theory, the findings suggest that continuous resource depletion arising from incompatible role demands reduces positive evaluations of academic work. The relatively strong effect observed in the present study further indicates that balancing professional and family responsibilities remains one of the most persistent challenges faced by female academics despite increasing institutional attention to employee wellbeing and work-life integration.

The findings also revealed that glass ceiling barriers significantly reduced job satisfaction. This result supports earlier studies suggesting that limited promotion opportunities, leadership exclusion, and organizational inequalities negatively influence employee attitudes and workplace outcomes (Cotter et al., 2001; Elacqua et al., 2009). Glass ceiling barriers were negatively associated with job satisfaction even though most respondents occupied permanent positions. Employment stability and career mobility should therefore be separated analytically. A secure appointment does not guarantee equal access to senior rank, strategic assignments, mentoring, leadership, or income progression. The result is more plausibly interpreted as a response to the anticipated return on professional investment than as a reaction to one promotion decision. When institutional procedures limit access to career resources, continued effort may be evaluated as less fair and less likely to produce recognition. This interpretation accords with research showing that academic inequality is reproduced through networks, selection routines, and standards of merit as well as through explicit exclusion (Acker and Wagner, 2019). More importantly, the findings are consistent with recent evidence indicating that gender-related structural barriers continue to constrain professional advancement and workplace wellbeing among women across various occupational settings (Patel et al., 2026; Wayne et al., 2019; Zhang et al., 2025). Although many higher education institutions formally endorse gender equality initiatives, the persistence of significant negative effects suggests that institutional commitments do not necessarily eliminate perceptions of career-related inequality. From a COR perspective, glass ceiling barriers may be interpreted as threats to future career resources, thereby reducing motivation and job satisfaction.

Technostress also had a substantial negative coefficient. Digital systems are often introduced as efficiency tools, but their effect depends on task design and governance. Multiple platforms, duplicated reporting, rapid changes, and communication outside working hours can increase workload without improving the substantive quality of teaching or research. The association is therefore not adequately explained by individual resistance to technology. It reflects the use of attention and recovery time by systems that require continued monitoring and adaptation. Findings among higher education teachers and researchers have similarly shown that technological demands can coexist with competence and still contribute to burnout and reduced wellbeing (Whelan et al., 2022; Zito et al., 2018). Similar findings have recently been reported in studies examining digital transformation, algorithmic work intensification, and technological disruption within professional environments (Ozkan and Balli, 2023; Özkan et al., 2025; Su and Jiang, 2023). The rapid digitalization of higher education has expanded expectations regarding online teaching, digital administration, virtual collaboration, and technological responsiveness. While technological advancement undoubtedly improves efficiency and accessibility, the present findings suggest that increasing technological complexity may simultaneously create psychological burdens that diminish employee wellbeing. Consequently, digital transformation should not be viewed solely as a source of organizational progress but also as a potential contributor to occupational strain when adequate support mechanisms are absent.

Perceived supportive organizational culture weakened each negative association, but the interaction coefficients were modest relative to the direct coefficients. This difference is theoretically important. The results support a buffering proposition, not a claim that supportive culture fully compensates for demanding work. Conditional slopes remained negative at high support, and the most favorable slope still indicated lower job satisfaction as each demand increased. The statistical evidence therefore supports a bounded rather than absolute version of the support paradox.

The buffering mechanism can be specified through resource preservation. Flexible arrangements may protect time when work and family requirements collide. Transparent communication, recognition, and career development may preserve procedural trust and access to professional resources when promotion systems are perceived as unequal. Technical help, advance communication, and digital boundary rules may reduce uncertainty and invasion when systems change. These resources alter how costly a demand becomes. They do not necessarily alter the number of tasks, redistribute leadership opportunities, remove biased procedures, or reduce the number of platforms in use.

Catholic institutional values sharpen this distinction. Human dignity, solidarity, and concern for employee development can strengthen interpersonal support and normative commitment, but values produce organizational consequences only when translated into enforceable procedures. A university may communicate care while retaining schedules that ignore care responsibilities, may recognize performance while leaving sponsorship networks unequal, or may promote employee wellbeing while normalizing after-hours digital responsiveness. The support paradox is observed at this implementation boundary: support is meaningful enough to attenuate adverse associations, yet insufficiently structural to neutralize their sources. The explanatory and predictive findings reinforce a restrained interpretation. *R*^2^ = 0.464 indicates that the integrated model captures a meaningful share of job satisfaction, but more than half of the variance was outside the model. PLSpredict further showed that positive predictive relevance did not translate into better error performance than the linear benchmark for most indicators. Interaction terms can improve theoretical interpretation without generating strong forecasts, particularly when omitted institutional and personal factors contribute to the outcome. External validation is therefore needed before the model is used to identify individuals or institutions at risk.

The findings provide important insights into what may be described as the support paradox. Although supportive organizational culture did not eliminate work–family conflict, glass ceiling barriers, or technostress, it significantly reduced their negative consequences for job satisfaction. This observation challenges simplistic assumptions that supportive organizational values alone are sufficient to eradicate workplace inequalities and occupational strain. Similar contradictions have recently been observed in organizations that formally promote employee wellbeing yet continue to expose employees to demanding work conditions and resource depletion processes (Demerouti, 2025a; Gonçalves et al., 2025). The present study extends this emerging line of research by demonstrating that supportive organizational culture functions as a buffering resource rather than a curative mechanism. Consequently, organizational support should be viewed as a means of mitigating institutional pressures rather than completely removing their underlying causes.

The study contributes to the JD-R and COR literature in several ways. First, the findings demonstrate that work–family conflict, glass ceiling barriers, and technostress should be understood collectively as multidimensional institutional demands rather than as isolated workplace stressors. Second, the study extends the buffering perspective of the JD-R framework by showing that supportive organizational culture simultaneously reduces the negative effects of multiple institutional pressures. Third, the findings contribute to the growing literature on employee wellbeing in faith-based higher education institutions by highlighting how organizational support shapes the experiences of female academics operating within value-driven organizational environments. The results suggest that organizational values and organizational practices do not necessarily generate equivalent outcomes, thereby reinforcing the importance of examining how institutional support is translated into everyday workplace experiences.

Three theoretical contributions are offered. First, work–family conflict, glass ceiling barriers, and technostress are integrated as multidimensional work-related demands rather than treated as interchangeable stressors. The distinction preserves the source of each resource loss: role incompatibility, constrained career access, and digitally intensified work. Second, supportive organizational culture is positioned as an individual perception of a contextual resource whose effect is conditional and demand-specific. This avoids a level-of-analysis error while retaining the JD–R proposition that resources can moderate demands. Third, the support paradox is delimited empirically. It is not inferred merely because support and strain coexist; it is demonstrated through significant attenuation combined with negative conditional slopes that remain after support increases. This formulation extends JD–R and COR theory by distinguishing protection from removal and by identifying the point at which cultural support must be converted into structural redesign.

Recommendations should be tied to the measured dimensions of supportive organizational culture. Organizational communication should be strengthened through published workload standards, accessible promotion criteria, clear explanations of digital changes, and regular reporting on gendered career outcomes. Employee recognition should cover teaching, research, service, administrative labor, mentoring, and work that sustains student welfare rather than reward only highly visible outputs. Recognition criteria should be documented so that appreciation does not depend on informal proximity to decision makers.

Work life flexibility should be implemented through meeting schedules within standard working hours, reasonable options for remote or adjusted work when duties permit, workload revision during intensive caregiving periods, and institutional rules that distinguish urgent from non-urgent digital communication. Career development support should include structured mentoring and sponsorship, transparent access to leadership assignments, assistance with promotion portfolios, research time protection, and cross-institutional networks for female academics. Commitment to employees should be made auditable through periodic workload reviews, technical-support standards, wellbeing services, pay-equity and promotion audits, and corrective plans when unequal outcomes persist.

Responsibility extends beyond individual universities. APTIK can establish shared indicators for female representation, time to promotion, workload distribution, and access to career development across member institutions. Higher education regulators can require gender-disaggregated reporting and encourage institutions to evaluate the human consequences of digital administrative systems. Researchers should avoid treating supportive culture as a general remedy and should identify which resource matches each demand. Support that is relational may be valuable but will remain limited when the primary source of strain is structural or technological.

Several limitations constrain the conclusions. The cross-sectional design does not establish temporal or causal order. Lower job satisfaction may also influence the reporting of work demands and organizational support. Longitudinal research should test whether changes in workload, promotion procedures, digital systems, and perceived culture precede changes in satisfaction. Quasi-experimental evaluations of specific institutional reforms would provide stronger evidence than repeated perception surveys alone.

All variables were obtained from a single self-report questionnaire. Procedural safeguards and low full-collinearity VIF values reduce concern about severe common method inflation but do not remove it. Future studies should combine survey responses with workload records, digital communication logs, promotion data, leadership representation, and independent assessments of institutional policy. Temporal separation of predictors and outcomes would also strengthen measurement design. The sampling procedure was criterion-based and nonrandom. Respondents were distributed across 22 institutions, but five institutions contributed 51.7% of the cases. Institutional clustering was not modeled, and standard errors may therefore be optimistic if responses within the same university were correlated. A multilevel design with sufficient institutions and respondents per institution should distinguish individual perceptions from institution-level culture and test cross-level moderation directly. Such a design would also permit institutional policies to be compared with employee perceptions.

The exceptionally high higher-order reliability and AVE values raise a further concern. Although the two-stage specification and homogeneous dimensions contribute to these estimates, redundant indicators or overly narrow dimension content cannot be excluded. Future validation should examine item-level descriptive statistics, residual correlations, content validity, measurement invariance, and the performance of shorter scales in independent samples. Mixed-method research would be useful for determining whether similarly worded items represent redundancy or substantively distinct experiences expressed through a shared organizational vocabulary.

Generalizability is limited to the sampled Catholic institutions and is strongest for permanent academics, respondents within the first 10 years of service, and those at assistant or associate ranks. Comparative studies should include secular universities, other religious traditions, different national systems, and male academics without treating sex as the only relevant difference. Interviews and institutional case studies could further examine how Catholic values are translated into workload, promotion, digital governance, and everyday support.

Work family conflict, glass ceiling barriers, and technostress were each negatively associated with the job satisfaction of female academics in Indonesian Catholic higher education. Perceived supportive organizational culture weakened these associations, but the moderation coefficients were smaller than the direct coefficients and the conditional effects remained negative at high support. The evidence therefore supports a limited buffering role rather than a curative interpretation of organizational culture. The support paradox is best understood as an implementation problem. Supportive values and practices can preserve resources and reduce the cost of demanding work, yet persistent role, career, and digital pressures require structural correction. Job satisfaction is unlikely to be sustained through symbolic commitments or interpersonal support alone. Greater protection will depend on whether communication, recognition, flexibility, career development, and commitment to employees are converted into transparent, equitable, and auditable organizational arrangements.

The study contributes to the Job Demands–Resources (JD-R) and Conservation of Resources (COR) literature by demonstrating that multidimensional institutional demands collectively undermine employee wellbeing, while supportive organizational culture serves as a buffering mechanism that reduces their negative impact. The findings further introduce the notion of the support paradox, whereby supportive organizational environments continue to coexist with substantial workplace pressures experienced by female academics. From a practical perspective, universities should move beyond symbolic commitments to employee wellbeing by implementing gender-sensitive workload policies, equitable career advancement systems, healthier digital work practices, and institutional support mechanisms that directly address the challenges faced by female academics. Such initiatives are essential for fostering sustainable academic careers, improving job satisfaction, and creating more inclusive higher education environments.

## Data Availability

The raw data supporting the conclusions of this article will be made available by the authors, without undue reservation.
