# Supplementary material for: The support paradox: work family conflict, glass ceiling barriers, technostress, and job satisfaction among female academics in Indonesian Catholic higher education
Source: Front Sociol. 2026 Sep 4;11:1907341. doi: 10.3389/fsoc.2026.1907341 (PMC13585508; doi:10.3389/fsoc.2026.1907341)
Supplement: Supplementary file 1 [file Data_Sheet_1.PDF]

SUPPLEMENTARY MATERIAL

The Support Paradox: Work-Family Conflict, Glass Ceiling Barriers, Technostress, and Job Satisfaction among Female Academics in Indonesian Catholic Higher Education

Ega Leovani, Mohamad Adam, and Muhammad Ichsan Hadjri

Detailed SmartPLS 4 Measurement and Structural Model Results

This supplementary file reports the original PLS algorithm output and the detailed measurement- and structural-model statistics for 406 female academics. Values were extracted from the authors' SmartPLS 4 report. Bootstrapping significance tests were based on 5,000 resamples.

Original SmartPLS output

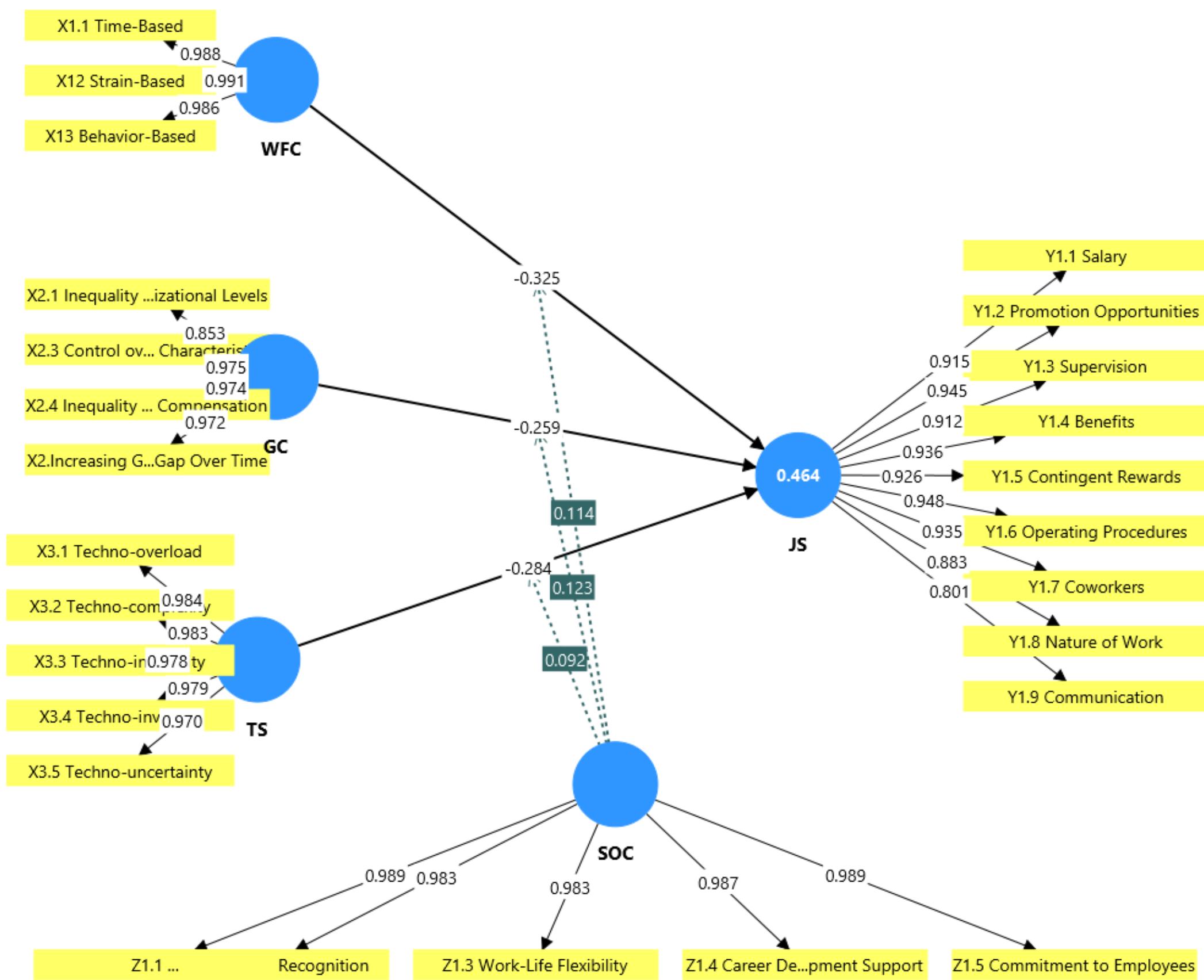

Supplementary Figure S1. Original SmartPLS PLS algorithm output.

Note. *Numbers beside the measurement paths are outer loadings; numbers on the structural paths are standardized path coefficients; the value inside job satisfaction is R^2. WFC = work-family conflict; GC = glass ceiling barriers; TS = technostress; SOC = perceived supportive organizational culture; JS = job satisfaction.*

Measurement model results

Supplementary Table S1. Outer loadings of the higher-order construct dimensions.

Construct	Dimension/indicator	Outer loading
**Work-family conflict**	X1.1 Time-Based	0.988
**Work-family conflict**	X1.2 Strain-Based	0.991
**Work-family conflict**	X1.3 Behavior-Based	0.986
**Glass ceiling barriers**	X2.1 Inequality at Higher Organizational Levels	0.853
**Glass ceiling barriers**	X2.2 Control over Job Characteristics	0.975
**Glass ceiling barriers**	X2.3 Inequality in Promotion and Compensation	0.974
**Glass ceiling barriers**	X2.4 Increasing Gender Gap over Time	0.972
**Technostress**	X3.1 Techno-overload	0.984
**Technostress**	X3.2 Techno-complexity	0.983
**Technostress**	X3.3 Techno-insecurity	0.978
**Technostress**	X3.4 Techno-invasion	0.979
**Technostress**	X3.5 Techno-uncertainty	0.970
**Job satisfaction**	Y1.1 Salary	0.915
**Job satisfaction**	Y1.2 Promotion Opportunities	0.945
**Job satisfaction**	Y1.3 Supervision	0.912
**Job satisfaction**	Y1.4 Benefits	0.936
**Job satisfaction**	Y1.5 Contingent Rewards	0.926
**Job satisfaction**	Y1.6 Operating Procedures	0.948
**Job satisfaction**	Y1.7 Coworkers	0.935
**Job satisfaction**	Y1.8 Nature of Work	0.883
**Job satisfaction**	Y1.9 Communication	0.801
**Perceived supportive organizational culture**	Z1.1 Organizational Communication	0.989
**Perceived supportive organizational culture**	Z1.2 Employee Recognition	0.983
**Perceived supportive organizational culture**	Z1.3 Work-Life Flexibility	0.983
**Perceived supportive organizational culture**	Z1.4 Career Development Support	0.987
**Perceived supportive organizational culture**	Z1.5 Commitment to Employees	0.989

Note. *All reported loadings exceed 0.70. The interaction constructs are single product terms with loadings fixed at 1.000 and are therefore not listed as substantive measurement indicators.*

Supplementary Table S2. Construct reliability and convergent validity.

Construct	Cronbach's alpha	rho_A	rho_C	AVE
**Glass ceiling barriers**	0.959	0.969	0.971	0.893
**Job satisfaction**	0.974	0.976	0.978	0.832
**Perceived supportive organizational culture**	0.993	1.002	0.994	0.972
**Technostress**	0.989	0.990	0.991	0.958
**Work-family conflict**	0.988	0.989	0.992	0.977

Note. *rho_A for perceived supportive organizational culture slightly exceeds 1.000 because it is a consistent reliability estimate derived from highly correlated higher-order dimension scores. The unusually high reliability and AVE values should be interpreted cautiously because they may indicate conceptual overlap or indicator redundancy.*

Discriminant validity

Supplementary Table S3. Heterotrait-monotrait ratio (HTMT).

Construct	GC	JS	SOC	TS	WFC
**GC**	-	0.356	0.167	0.106	0.040
**JS**	0.356	-	0.337	0.404	0.405
**SOC**	0.167	0.337	-	0.165	0.058
**TS**	0.106	0.404	0.165	-	0.089
**WFC**	0.040	0.405	0.058	0.089	-

Note. *All HTMT values are below 0.85. GC = glass ceiling barriers; JS = job satisfaction; SOC = perceived supportive organizational culture; TS = technostress; WFC = work-family conflict.*

Supplementary Table S4. Fornell-Larcker criterion.

Construct	GC	JS	SOC	TS	WFC
**GC**	0.945	-0.346	-0.165	0.104	0.039
**JS**	-0.346	0.912	0.336	-0.397	-0.398
**SOC**	-0.165	0.336	0.986	-0.165	-0.059
**TS**	0.104	-0.397	-0.165	0.979	0.089
**WFC**	0.039	-0.398	-0.059	0.089	0.989

Note. *Diagonal values are the square roots of AVE; off-diagonal values are latent-variable correlations. Each diagonal value exceeds the corresponding inter-construct correlations.*

Structural model results

Supplementary Table S5. Structural path coefficients, bootstrapping results, effect sizes, and collinearity.

Hyp.	Structural relationship	beta	t	p	95% BC CI	f²	VIF	Decision
**H1**	Work-family conflict -> Job satisfaction	-0.325	8.864	<0.001	[-0.392, -0.249]	0.190	1.038	Supported
**H2**	Glass ceiling barriers -> Job satisfaction	-0.259	6.899	<0.001	[-0.330, -0.183]	0.118	1.058	Supported
**H3**	Technostress -> Job satisfaction	-0.284	7.902	<0.001	[-0.354, -0.213]	0.141	1.069	Supported
**-**	Perceived supportive organizational culture -> Job satisfaction	0.216	5.463	<0.001	[0.138, 0.294]	0.083	1.056	Main effect
**H4**	SOC x Work-family conflict -> Job satisfaction	0.114	2.747	0.006	[0.028, 0.192]	0.023	1.044	Supported
**H5**	SOC x Glass ceiling barriers -> Job satisfaction	0.123	2.956	0.003	[0.040, 0.202]	0.026	1.034	Supported
**H6**	SOC x Technostress -> Job satisfaction	0.092	2.339	0.019	[0.016, 0.172]	0.015	1.050	Supported

Note. *BC CI = bias-corrected confidence interval. Bootstrapping used 5,000 resamples. For f², values around 0.02, 0.15, and 0.35 are commonly interpreted as small, medium, and large. All inner-model VIF values are close to 1.00 and indicate no problematic structural collinearity.*

Supplementary Table S6. Conditional direct effects at different levels of perceived supportive organizational culture.

Relationship	SOC at -1 SD	SOC at mean	SOC at +1 SD
**Work-family conflict -> Job satisfaction**	-0.439	-0.325	-0.211
**Glass ceiling barriers -> Job satisfaction**	-0.382	-0.259	-0.137
**Technostress -> Job satisfaction**	-0.376	-0.284	-0.192

Note. *The negative slopes become less negative as perceived supportive organizational culture increases, which is consistent with a buffering effect. However, all conditional slopes remain negative.*

Model explanatory power and fit

Supplementary Table S7. Explanatory power and model-fit statistics.

Criterion	Saturated model	Estimated model
**R^2 for job satisfaction**	-	0.464
**Adjusted R^2 for job satisfaction**	-	0.454
**SRMR**	0.035	0.035
**d_ULS**	0.419	0.422
**d_G**	1.264	1.255
**Chi-square**	2518.556	2521.351
**NFI**	0.881	0.881

Note. *R^2 and adjusted R^2 apply to job satisfaction and are displayed in the estimated-model column. The SRMR value is below 0.08. Model-fit indices are reported as descriptive diagnostics and should be interpreted together with predictive and substantive assessment.*

Abbreviations and source

GC = glass ceiling barriers; JS = job satisfaction; SOC = perceived supportive organizational culture; TS = technostress; WFC = work-family conflict; AVE = average variance extracted; VIF = variance inflation factor; SRMR = standardized root mean square residual; NFI = normed fit index.

Source: Authors' analysis in SmartPLS 4 using the uploaded SmartPLS report "PLS Algoritm moderasi English(1).xlsx".
